# Influence of Chain Stiffness, Grafting Density and Normal Load on the Tribological and Structural Behavior of Polymer Brushes: A Nonequilibrium-Molecular-Dynamics Study

**DOI:** 10.3390/polym8070254

**Published:** 2016-07-08

**Authors:** Manjesh K. Singh, Patrick Ilg, Rosa M. Espinosa-Marzal, Nicholas D. Spencer, Martin Kröger

**Affiliations:** 1 Laboratory for Surface Science and Technology, Department of Materials, ETH Zurich, 8093 Zurich, Switzerland; manjesh.singh@mat.ethz.ch; 2 School of Mathematical and Physical Sciences, University of Reading, Reading RG6 6AX, UK; p.ilg@reading.ac.uk; 3 Laboratory for Smart Interfaces in Environmental Nanotechnology, Department of Civil and Environmental Engineering, University of Illinois at Urbana-Champaign, Champaign, IL 61801, USA; rosae@illinois.edu; 4 Polymer Physics, Department of Materials, ETH Zurich, CH–8093 Zurich, Switzerland

**Keywords:** boundary lubrication, shear, coefficient of friction, solvent quality, semiflexible polymers, planar brush, bending stiffness, FENE-chain model, polymer brushes

## Abstract

We have performed coarse-grained molecular-dynamics simulations on both flexible and semiflexible multi-bead-spring model polymer brushes in the presence of explicit solvent particles, to explore their tribological and structural behaviors. The effect of stiffness and tethering density on equilibrium-brush height is seen to be well reproduced within a Flory-type theory. After discussing the equilibrium behavior of the model brushes, we first study the shearing behavior of flexible chains at different grafting densities covering brush and mushroom regimes. Next, we focus on the effect of chain stiffness on the tribological behavior of polymer brushes. The tribological properties are interpreted by means of the simultaneously recorded density profiles. We find that the friction coefficient decreases with increasing persistence length, both in velocity and separation-dependency studies, over the stiffness range explored in this work.

## 1. Introduction

End-grafted polymer chains form a brush-like structure when they are grafted sufficiently close to each other on a substrate. Such “polymer brushes”, due to their tunable behavior in different solvents, find application in the fields of colloidal stabilization, protein adsorption and tribology [1,2,3,4,5]. A large number of experimental [6,7,8,9,10,11] and computational [12,13,14,15,16,17,18,19,20,21,22] studies on the tribological behavior of various polymer brushes in different solvents under various controlled conditions had been performed. The experimental studies have been carried using atomic force microscopy (AFM) [23,24,25,26] and the surface-forces apparatus (SFA) [7,8,9,27]. Tsuji et al. [24,25] studied the tribological behavior of concentrated polymer brushes (CPBs) in mixtures of solvents. The effect of grafting density on the tribological response of polymer brushes was also studied, and a “transition-load” was reported at different grafting densities by Rosenberg et al. [26]. Pioneering theoretical work on polymer brushes is due to Alexander [28], de Gennes [29] and Semenov [30]. Alexander and de Gennes assumed a “step-like” density profile in their original contributions. Milner et al. [31] in their work later suggested a parabolic profile of the chemical potential that implies a parabolic density profile for brushes subjected to good solvents. Milner et al. [31] and Zhulina et al. [32] independently implemented the concept of strong stretching of brushes. They concluded that the brush height is typically much larger than the unperturbed linear size of a single chain, and a large amount of subsequent literature addressed analytically tractable scaling regimes under different conditions. Binder and Milchev [33] and Kreer [34] have reviewed the use of numerical calculations to study polymer brushes. Monte Carlo [35,36,37], Brownian-dynamics [38] and dissipative-particle-dynamics [39] approaches have been used extensively in computational studies of polymer brushes. Most of the molecular-dynamics (MD) studies of coarse-grained polymer brush systems have been performed using an implicit solvent [12,13,17,18,19,40]. In such an approach, the properties of the solvent, such as its velocity profile, are assumed rather than calculated. While implicit solvent-based MD simulations have obvious advantages when it comes to the computational cost, it also comes with many limitations. Implicit solvent-based MD simulations do not capture hydrodynamic effects well. Most of the existing explicit solvent-based MD simulations of polymer brushes have focused on brush profiles and other static properties for flexible chains. Galuschko et al. [20] and Dimitrov et al. [41] used dissipative-particle dynamics (DPD) as a thermostat in their simulation studies. The MD simulation study of Galuschko et al. [20] compared results from implicit solvent and explicit solvent-based approaches. Simulations were performed at different shear velocities, keeping the separation constant and also for different grafting densities. Dimitrov et al. [41] investigated explicit solvent-based MD simulations to study the influence of solvent quality on the structure and thermodynamic properties of end-grafted polymer chains. Friction between ring polymer brushes at melt densities was studied by Erbas and Paturej [42] and compared to the results for friction between linear polymer brushes. The work shows that both friction force and normal pressures were greater by a factor of two for the linear polymer brushes in comparison to ring polymer brushes. This leads to a similar coefficient of friction in both cases, but the effective viscosity is found to be different. They also observed enhanced interpenetration between linear polymer brushes compared to their circular counterparts. Carrillo et al. [43,44] studied and compared friction between charged and neutral bottle brushes via MD. They varied the shear stress and compression of confined bilayers to study the effect of grafting density and shear rate on the deformation, viscosity, and friction behavior. Speyer and Pastorino [45] studied the influence of grafting density and chain stiffness on the friction behavior of polymer brushes in a super-hydrophobic regime in which the solvent did not penetrate the polymer brush. Atomistic MD techniques were used to study the tribology of other friction modifiers, as well. Doig et al. [46] and Ewen et al. [47] used MD simulations to investigate and interpret the tribological behavior of organic friction modifiers, whereas Lorenz et al. [48] studied self-assembled monolayers. To the best of our knowledge, no simulation studies were yet performed to reveal the effect of the normal load/separation (*D*), grafting density (*ρ*) and semiflexibility of chains in both brush and mushroom regimes where solvent penetrated into the tethered layer.

Here, we describe explicit solvent-based MD simulations on polymer brushes, investigating the effect of different parameters on the tribological behavior of polymer brushes. We describe in Section 2 our model approach and simulation details. Results and discussions in Section 3 begin with equilibrium MD simulations, reporting the radius of gyration ($R_{g}$) of a single chain, brush height (*h*) and normal stress ($\sigma_{zz}$) against separation distance (*D*) for flexible and semiflexible model polymer brushes. Next, we present the tribological behavior of uncompressed, marginally compressed and compressed brush bilayers over a range of shear velocities (*v*). We then study the effect of grafting density (*ρ*) on the shear behavior of flexible brushes. Further, we explore the effect of chain stiffness ($k_{b}$) on the tribological behavior of polymer brushes. Finally, to separate the effect of stiffness, we have carefully selected two pairs of grafting-density and chain-stiffness values, resulting in a comparable equilibrium-brush height and then have studied the tribological response of the two sets. We conclude with a summary of current findings and the outlook for prospective work.

## 2. Model

### 2.1. Connectivity

**Figure 1:**
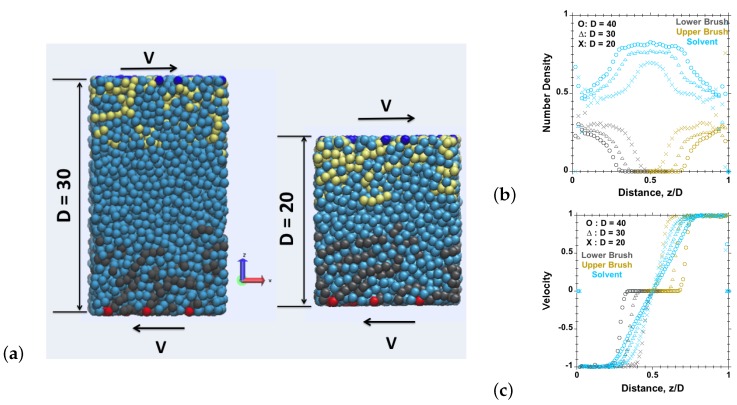
Representative information from a brush-against-brush system with explicit solvent, subjected to shear. (**a**) Snapshots, where polymer beads are colored by black (bottom) and yellow (top), tethered beads colored by red (bottom) and blue (top) and solvent beads colored by cyan; (**b**) number density profiles and (**c**) velocity profiles versus distance to the bottom surface (M=20 chains tethered on each surface, N=30 beads per chain, surface-tethering density ρ=0.075, at velocity v=1 applied on tethered beads on both sides in opposite directions at separation D=50). All dimensional quantities are given in Lennard–Jones (LJ) units. These particular sets of simulations were performed at very high shear velocity, v=1, to achieve a visible amount of alignment, whereas the shear velocity of all subsequent simulations does not exceed v=0.05.

A linear chain consisting of *N* beads represents each polymer chain within the polymer-brush system. Each bead in the chain may be representing more than a single monomer of a real polymer chain. Adjacent beads in each chain are bonded using a finite extendable nonlinear elastic (FENE) potential [15]. Each chain is attached by one of its ends to the substrate using a permanently tethered bead (red and blue beads in Figure 1), and the rest of the beads of each chain are free to move and interact with all other polymer and solvent beads via the $\varepsilon_{\mathrm{pp}}$ and $\varepsilon_{\mathrm{ps}}$ potentials specified below. Single spherical beads (cyan beads in Figure 1) represent the solvent particles. All of the simulations were performed for brush-against-brush systems in a planar setup. Periodic boundary conditions have been applied only along the axes parallel to the sliding surfaces.

### 2.2. Excluded Volume

The excluded volume and attractive interaction between polymer and solvent beads is modeled following Soddemann [49] and Dimitrov et al. [41] in their previous works. Within this model, the Lennard–Jones (LJ/12-6) potential is smoothly truncated at a cut-off distance $r_{c}=1.5$. This is achieved by using the LJ/12-6 potential only up to its minimum and shifting to some desired depth ($\varepsilon_{\mathrm{pp}}$, $\varepsilon_{\mathrm{ps}}$), continued from its minimum to zero with a potential having a cosine form. This provides a potential that is both continuous and also has a continuous derivative. Thus, at small distances, the model potential corresponds to the purely repulsive, so-called shifted Weeks–Chandler–Anderson (WCA) potential [50,51]:

(1) $$\begin{array}{ccc} U_{\mathrm{WCA}}\left(r\right) & = & 4\varepsilon_{\mathrm{LJ}}\left[{\left(\frac{\sigma_{\alpha \beta }}{r}\right)}^{12}-{\left(\frac{\sigma_{\alpha \beta }}{r}\right)}^{6}+\frac{1}{4}\right]-\varepsilon_{\alpha \beta },\;\;\frac{r}{\sigma_{\alpha \beta }}\leq \sqrt[{6}]{2} \end{array}$$

where the index (αβ) stands for the different types of pairs: polymer-polymer (pp), polymer-solvent (ps) or solvent-solvent (ss), and we choose the range parameters to be identical, $\sigma_{\mathrm{pp}}=\sigma_{\mathrm{ps}}=\sigma_{\mathrm{ss}}$. Scales for length and energy (and temperature) are chosen such that both $\sigma_{\mathrm{pp}}=1$ and $\varepsilon_{\mathrm{LJ}}=1$ (Boltzmann’s constant $k_{B}=1$). At intermediate distances, the interaction is purely attractive and parameterized by a cosine potential of the form:

(2) $$\begin{array}{ccc} U_{\mathrm{LJcos}}\left(r\right) & = & \frac{\varepsilon_{\alpha \beta }}{2}\left\{\cos\left[c_{1}{\left(r/\sigma_{\alpha \beta }\right)}^{2}+c_{2}\right]-1\right\},\;\;\sqrt[{6}]{2}\leq \frac{r}{\sigma_{\alpha \beta }}\leq \frac{3}{2} \end{array}$$

while $U_{\mathrm{LJcos}}\left(r\right)=0$ for larger distances. As explained in detail elsewhere [41], we have used a value of $c_{1}=3.173$ and $c_{2}=-0.856$ to ensure that the potential ($U_{\mathrm{LJcos}}$) and its derivative are continuous both at the potential minimum, $r/\sigma_{\alpha \beta }=\sqrt[{6}]{2}$, and at the potential cut-off, $r/\sigma_{\alpha \beta }=3/2$. For our simulations, we have used the purely repulsive potential for polymer-polymer ($\varepsilon_{\mathrm{pp}}=0$) and solvent-solvent interactions ($\varepsilon_{\mathrm{ss}}=0$), while the polymer-solvent interaction is modeled using $\varepsilon_{\mathrm{ps}}=0.4$. This combination ensures a good-solvent quality [41,52].

### 2.3. Temperature, Deformation and Wall

The temperature was maintained constant by rescaling *peculiar velocities* [53] of all, except tethered, beads. The macroscopic velocity due to shearing is thus taken into account in the definition of peculiar velocities. We have used a profile unbiased thermostatting (PUT) scheme where the center-of-mass velocity for each set of beads residing in layers parallel to the wall is computed and used to define the ‘bias’ velocity. This bias velocity is then subtracted from the velocities of individual beads to yield a thermal velocity for each bead. The thermal velocity is rescaled to the desired value, and subsequently, the bias velocity is added [53,54]. To ensure that polymer and solvent beads do not cross the grafting surface, a purely repulsive LJ/9-3 potential was used. The motivation for using a wall potential at the grafting surface was to reduce the computational cost. Another approach used in nonequilibrium-MD (NEMD) studies is to thermostat [21,22] the explicit wall, rather than the confined fluid. Bernardi et al. [55] have shown in their study that direct thermostatting of confined fluids under shear strongly influence their behavior. It is important to mention here that their study was carried out at a very large shear rate, $\dot{\gamma }=1.0$. In our study, we have applied shear rates orders of magnitude smaller with Weissenberg numbers well below unity (except at the maximum velocity, v=0.05). Direct thermostatting of the confined fluid in NEMD studies of polymer brushes is fairly common.

### 2.4. Semiflexibility

For chain-stiffness modeling, we have used the following discrete version of a bending potential:

(3) $$\begin{array}{c} \frac{U_{b}}{k_{B}T}=k_{b}\sum_{i=1}^{N-1}\left(1-u_{i}\cdot u_{i+1}\right) \end{array}$$

where $k_{b}$ is the dimensionless bending stiffness and $u_{i}$ denotes the unit segment vector connecting the *i*-th with the (i+1)-th bead along the polymer chain. The bending stiffness $k_{b}$ and persistence length $L_{p}$ are interrelated via $L_{p}=-b_{0}/\ln L\left(k_{b}\right)$ with a bond length $b_{0}\approx 1$, which can readily be estimated from the already stated intramolecular potential at the given temperature, and the Langevin function $L\left(k_{b}\right)=\coth\left(k_{b}\right)-1/k_{b}$ [56].

### 2.5. Simulation Details

The dynamics of such a molecular model at constant temperature were explored by solving modified (thermostatted) Newton’s equations with the explicit solvent. We have used LAMMPS (Large-scale Atomic/Molecular Massively Parallel Simulator) [57], an open-source code, for all simulation work. The calculated chain trajectories provide complete information about chain alignment, density profiles and the stress tensor. All simulated quantities reported in the current study are given in terms of LJ units [54,58].

We have carried out simulations for the brush-against-brush model system described in Figure 1. Three types of beads: non-tethered, tethered and solvent, were used. The simulations were performed on randomly grafted polymer chains on flat surfaces. The system consists of M=20 chains on each tethering surface, while each linear chain is composed of N=30 beads. It is important to mention here that the critical grafting density ($\rho^{*}=1/\pi R_{g}^{2}$) [20] for chains having N=30 beads is $\rho^{*}\approx 0.025$ (in LJ units). We have considered four grafting densities: ρ=0.015 (less than critical grafting density), ρ=0.025 (equal to critical grafting density), ρ=0.075 (three-times the critical grafting density) and ρ=0.15 (six-times the critical grafting density). The simulations were carried out for different values of chain stiffness, $k_{b}=0$, 1 and 3. The total number of beads in the simulation box is such that the number density of all beads is approximately 0.8 at each separation between walls.

We equilibrated all systems carefully first with $5\times 10^{5}$ integration steps with Δt=0.001, followed by $6\times 10^{6}$ and $10^{7}$ steps with Δt=0.002 for ρ=0.075, 0.15 and ρ=0.015, 0.025, respectively. Data were then extracted by additional $10^{7}$ time steps with Δt=0.0025. A reduction of separation distance by one (LJ unit) was achieved as follows. An amount of solvent beads compatible with unchanged number density 0.8 at the new separation distance was randomly removed from the system. One grafting surface was kept fixed and the other was moved with a constant velocity, v=0.01, for a duration of 100,000 steps at Δt=0.001. At each separation *D* between the polymer-chain-bearing surfaces, the polymer-brush system was allowed to equilibrate for $3\times 10^{6}$ time steps ($10^{6}$ steps at Δt=0.001 followed by $2\times 10^{6}$ steps at Δt=0.0025).

These equilibrated systems were used to run NEMD simulations. Steady shear was applied by moving the tethered beads on both sides with the same prescribed velocity in opposite directions, keeping the separation constant during each run of given shear velocity [59]. At each separation and velocity, the stress tensor was calculated using the Irving–Kirkwood expression [54,58,60]. The temperature was maintained constant at T=1.2 using peculiar-velocity rescaling as discussed in the previous section. We have performed NEMD simulations keeping the separation between grafting surfaces constant. The Gattinoni et al. [61] study of confined fluids showed that a constant separation between walls may lead to results that differ from those obtained at constant load, especially at small wall separations. Experimental studies of the tribological behavior of polymer brushes have been performed subjected to both constant wall separation [7,8,9,27] and constant load [23,24,62]. For the present work, we have chosen to operate at constant separation and measured the load, rather than the opposite.

Two kinds of studies were carried out: (i) separation-dependent: different separations while maintaining a fixed velocity (Table 1) and (ii) velocity-dependent: different velocities while keeping the separation between tethering surfaces constant (Table 2). For the velocity-dependency studies, the simulations were carried out over a range of shear velocities v=0.00015–0.05 at fixed separations. At all velocities, data during the first 500,000 time steps (Δt=0.001) belonging to the startup phase were ignored for the analysis of stationary quantities. Subsequently, the simulations were performed for different numbers of time steps at different velocities with Δt=0.0025. At the maximum velocity v=0.05, simulations were run for 200,000 time steps following a startup period comprising half of this period of time. Similarly, at the slowest velocity v=0.00015, simulations were run for $7\times 10^{7}$ time steps following the startup phase of $3.5\times 10^{7}$ time steps. Simulations for each separation (*D*) and velocity (*v*) were repeated using 10 different initial configurations of randomly grafted polymer chains. The mean values from these runs are reported with standard deviations in Section 3.

## 3. Results and Discussion

### 3.1. Equilibrium

The equilibrium-brush height *h* of a single brush layer we define by the normal distance from the grafting surface where the brush begins to interact with the opposing brush, as seen from the time-averaged density profile. To be precise, we define *h* where the density profile falls below 1% of its maximum value close to the tethering surface. Using this criterion, the opposing brushes basically do not interact for D≥2h. Other definitions using moments of the density profile are certainly possible and common, as well.

**Figure 2:**
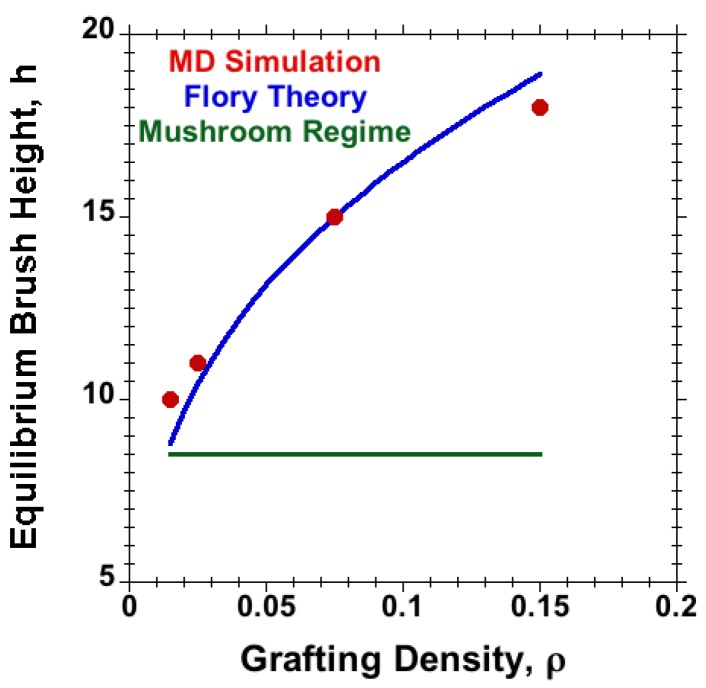
Equilibrium-brush height (*h*) versus grafting density (*ρ*) for M=20 flexible chains with N=30 under good-solvent conditions. The *ρ*-independent reference result for the mushroom regime is marked by the horizontal line. Flory results according to Section 3.3.

**Figure 3:**
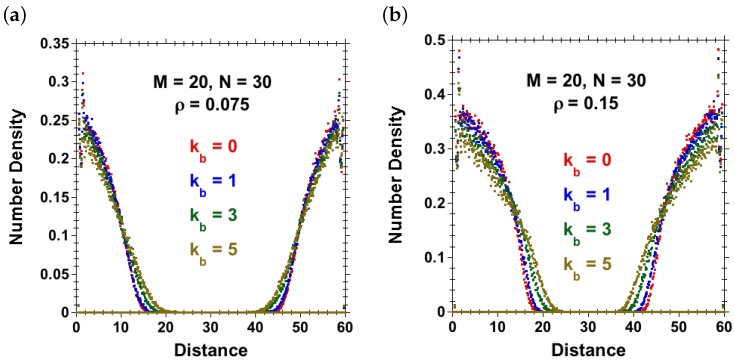
Polymer concentration profiles for non-interacting, opposing brushes at two grafting densities (**a**) ρ=0.075 and (**b**) ρ=0.15 for chains of various values of bending stiffness $k_{b}$ (M=30 chains with N=30 on each side). Brush heights shown in Figure 4 are extracted from these profiles.

Figure 2 shows the effect of grafting density on the equilibrium-brush height (*h*) of flexible polymers as calculated from the density profiles for each different grafting density at a large separation between the brush-bearing surfaces, while Figure 3 shows the density profiles for both flexible and semiflexible polymer-brush systems when the chains grafted on opposite surfaces were not in contact with each other. These density profiles were used to calculate and study the effect of chain stiffness ($k_{b}$) on the equilibrium-brush height (*h*).

**Figure 4:**
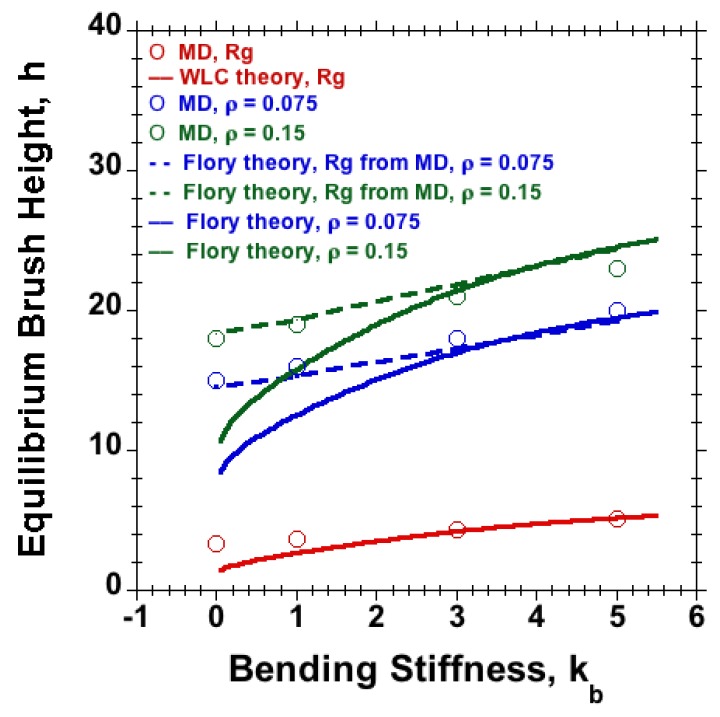
Radius of gyration $R_{g}$ of the free chain (red circles) and equilibrium-brush height versus chain bending stiffness ($k_{b}$) at two different grafting densities, ρ=0.075 (blue circles) and ρ=0.15 (green circles), obtained via MD for M=20 and N=30 under good-solvent conditions. For comparison, we show $R_{g}$ for the worm-like chain (WLC) model (solid red line), the Flory theory prediction for the brush height, Equation (4) with τ=1, using the radius of gyration $R_{g}$ according to the WLC model (solid blue and green lines for two different surface densities) or the $R_{g}$ of the free chain obtained via MD (dashed blue and green) as the undisturbed size $R_{0}$. While the Flory predictions are in excellent agreement with our MD data when the calculated equilibrium size of the polymer is used, the WLC naturally fails to capture the effect of excluded volume on the size and brush height in the limit of flexible chains ($k_{b}=0$).

Equilibrium-brush height (*h*) against chain stiffness ($k_{b}$) is plotted for brushes at grafting densities ρ=0.075 and ρ=0.15 in Figure 4. The results reveal an increase in the equilibrium-brush height with increasing chain stiffness for different grafting densities in agreement with the experiments of polymer brushes in solvent mixtures [63]. Following [64], for a planar brush under good-solvent conditions with $L\gg L_{p}$, one expects for the equilibrium-brush height within a Flory-type theory:

(4) $$\begin{array}{c} h≃{\left(\frac{\tau b_{0}\rho R_{0}^{2}}{L}\right)}^{1/3}L, \end{array}$$

where $L=\left(N-1\right)b_{0}$ is the length of the fully extended polymer chain, $b_{0}$ the mean bond length, *ρ* the tethering density and *τ* a dimensionless solvent-quality parameter, i.e., a positive $k_{b}$-independent constant of order unity under good-solvent conditions, and $R_{0}$ is the undisturbed equilibrium size of the free, semiflexible polymer. In the absence of excluded-volume interactions, our multi-bead chain with dimensionless bending stiffness $k_{b}$ should exhibit the statistical properties of a classical worm-like chain (WLC), characterized by mean square end-to-end distance $R_{\mathrm{ee}}^{2}=2L_{p}L\left\{1-L_{p}\left(1-\exp\left(-L/L_{p}\right)\right]/L\right\}$ and squared radius of gyration $R_{g}^{2}=L_{p}\left\{L/3-L_{p}+2\left(L_{p}^{2}/L^{2}\right)\left[L-L_{p}\left(1-e^{-L/L_{p}}\right)\right]\right\}$, where the persistence length $L_{p}$ is related to the bending stiffness $k_{b}$ via the before-mentioned relationship $L_{p}=-b_{0}/\ln L\left(k_{b}\right)$. For the comparison with the MD data, it does not qualitatively matter if we use $R_{\mathrm{ee}}$ or $R_{g}$ as the undisturbed equilibrium size $R_{0}$ in Equation (4). We use the latter in Figure 4 with τ=1 and an overall prefactor of order unity (1.68) to fit the data. While the Flory predictions are in excellent agreement with our MD data when the calculated equilibrium size of the polymer is used, the WLC naturally fails to capture the effect of excluded volume on the size and brush height in the limit of flexible chains ($k_{b}=0$), because the flexible FENE chain model has already a finite $L_{p}$ for $k_{b}=0$. The Flory-type result in Equation (4) follows from minimization of the free energy of a single chain, $F/k_{B}T=h^{2}/R_{0}^{2}+\tau N\phi$, with respect to *h*, where $\phi =Nb_{0}^{3}\rho /h$ denotes the *h*-dependent volume fraction within the planar brush. The limitations of the Flory approach were discussed in detail in [64]. Within the mushroom regime, where polymers do not overlap, the height *h* of the polymer layer should become independent of *ρ* and scale like the radius of a polymer, for which Flory theory predicts $h≃{\left(\tau b_{0}L^{2}R_{0}^{2}\right)}^{1/5}$. This corresponds to employing $\phi =Nb_{0}^{3}/h^{3}$ in the free energy expression.

**Figure 5:**
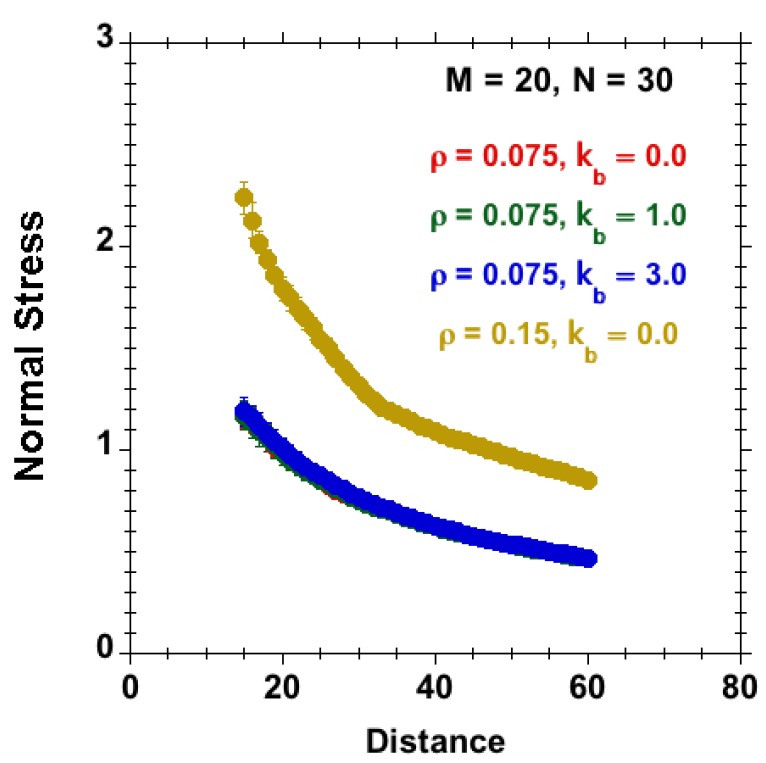
Normal stress versus gap size (system with M=20, N=30 subject to good-solvent conditions, constant bead number density) for different bending stiffness and surface densities, $k_{b}=0$ (red), $k_{b}=1$ (green) and $k_{b}=3$ (blue), for a brush system at ρ=0.075 and $k_{b}=0$ (yellow) for a more densely grafted system at ρ=0.15. The results are from equilibrium MD simulations. The normal stress is basically unaffected by bending stiffness over the shown range of distances.

The equilibrium behavior of the model brush in the absence of shear is characterized by the normal stress as a function of separation between opposing tethering surfaces. In Figure 5, we highlight both the significant effect of surface density on the normal stress and the apparent insensitivity of the normal stress on the intrinsic stiffness of chains. Note that tethering of chains does not prefer a particular orientation of the anchored segments. The equilibrium behavior is of relevance to rate the nonequilibrium behavior to be presented in the subsequent sections.

### 3.2. Sheared Brushes Made of Flexible Chains

#### 3.2.1. Effect of Shear Velocity at Various Separations, but Fixed Tethering Density

**Figure 6:**
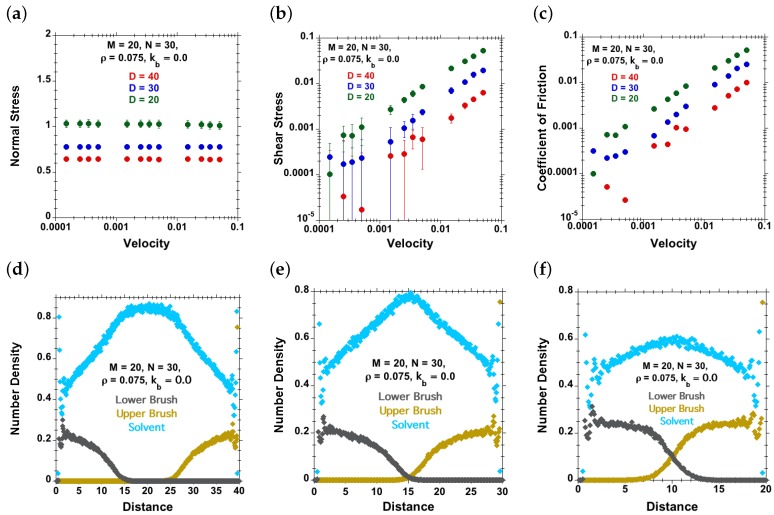
Effect of separation distance *D* (red dots for D=40, blue dots for D=30 and green dots for D=20) for symmetric brushes under good-solvent conditions, carrying M=20 chains on each tethering surface. Each linear, flexible ($k_{b}=0$) chain has N=30 beads, and the grafting density is ρ=0.075. (**a**) Normal stress against velocity; (**b**) shear stress against velocity; (**c**) coefficient of friction against velocity. Density profiles at shear velocity, v=0 of solvent (cyan dots), lower polymer-chains (grey dots) and upper polymer-chains (brown dots) at (**d**) D=40, (**e**) D=30 and (**f**) D=20.

In Figure 6, the shear and normal stresses are plotted for flexible chains ($k_{b}=0$) against shear velocity at different separations D=40 (uncompressed brushes), D=30 (marginally compressed brushes) and D=20 (highly compressed brushes). The normal stress is found to remain constant at all velocities at each separation, but increases with increasing brush compression. We observed an increase in friction (shear) forces with increasing compression, i.e., increasing normal load at all investigated velocities. Similar trends were observed in previous experimental studies [10,24,25]. The coefficients of friction (ratio between shear and normal stress) also increase with increasing velocity and with increasing compression. Figure 6 further shows the density profiles at the corresponding separations for uncompressed brushes, marginally compressed brushes and highly compressed brushes, where interpenetration is observed. At a separation of D=40, the grafted opposite polymer chains are not interacting with each other, and there is a layer of solvent in between the polymer outer surfaces, leading to a tribological behavior that is dominated by the friction behavior of the solvent, implying a very low or immeasurable friction coefficient [23]. As the surfaces are brought closer, the opposite chains interact with each other, and the coefficient of friction increases. With further compression, the penetration between opposing chains increases, resulting in a further increase in friction.

#### 3.2.2. Effect of Grafting Density and Separation at Fixed Velocity

Here, we have run the simulations at different separations *D* between polymer-bearing surfaces at a constant velocity, v=0.001, applied in opposing directions on tethered beads on both sides. Brush-against-brush systems were studied at various grafting densities, ρ=0.015, 0.025, 0.075 and 0.15 (LJ units) for a fixed M=20 and N=30. As discussed, the critical grafting density for linear chains having *N* = 30 beads is 0.025. We hence probed the mushroom regime (ρ=0.015), the system at critical grafting density, ρ=0.025, and the brush regime (ρ=0.075 and 0.15). The separation *D* was varied for each density, such that h<D<2h for the respective case, i.e., over a similar compression.

**Figure 7:**
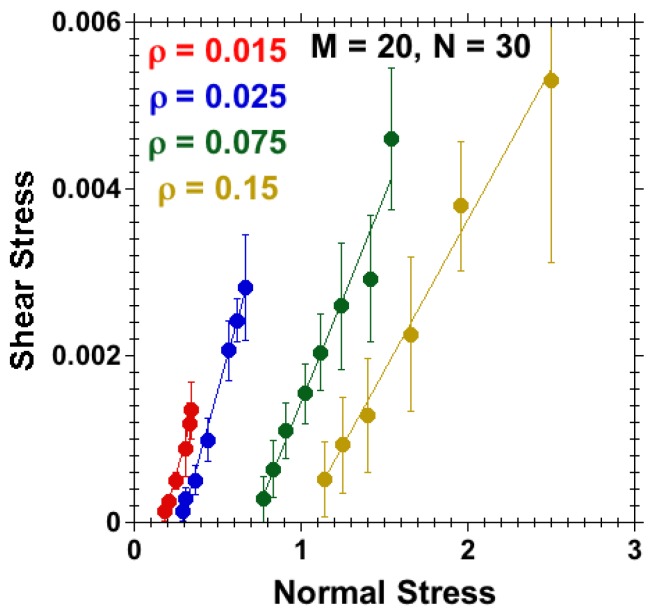
Effect of grafting density for M=20, N=30, $k_{b}=0$ and good-solvent quality; shear stress against normal stress for systems with grafting densities ρ=0.015, 0.025, 0.075 and 0.15. The stresses were calculated over a range of separations, *D*, such that D<2h for each data point. Straight lines represent linearly interpolated fits; their slopes are shown in Figure 8.

**Figure 8:**
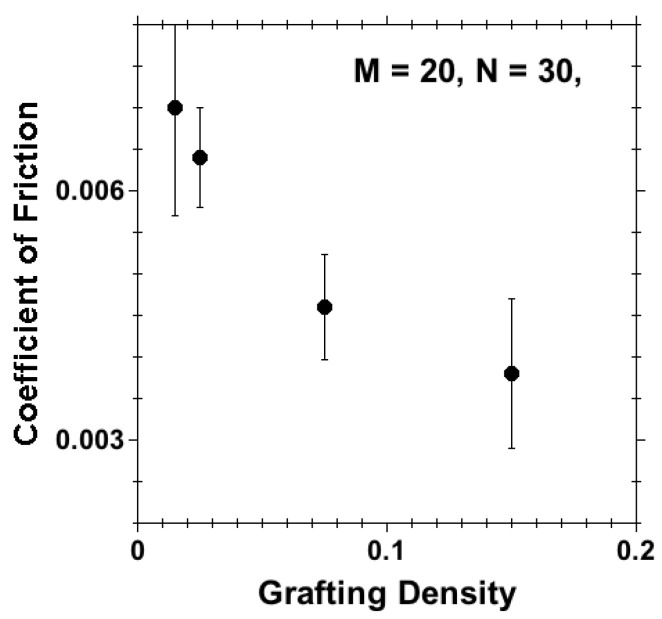
Coefficient of friction versus grafting density (*ρ*) for M=20, N=30 in the presence of a good solvent. Here, the coefficient of friction is calculated from the slope of shear stress against normal stress in Figure 7 (so-called Approach A) and not for a particular velocity (so-called Approach B).

The stresses calculated at different grafting densities are quantitatively very different. Figure 7 summarizes the results for for shear stress $\sigma_{xz}$ against normal stress $\sigma_{zz}$ calculated for different separations for systems with surface densities ρ=0.015, 0.025, 0.075 and 0.15, upon applying a shear velocity v=0.001 in opposing directions on tethered beads on each wall. The shear stress against normal stress data exhibit a linear dependency. Fitting a straight line (considering the error in each value) [65] to each curve, the coefficient of friction was calculated from the slope of the curve, as was also done in experiments [23]. The coefficient of friction thus obtained from the slope of the curves is plotted against the corresponding grafting densities in Figure 8.

We observe a decrease in the coefficient of friction with increasing grafting density, while this coefficient tends to become insensitive to grafting density within the brush regime. There is a stark decrease of the coefficient of friction when we enter from the mushroom regime (ρ=0.015) into the brush regime (ρ=0.075 and ρ=0.15). This can be explained as follows. With the increase in grafting density (*ρ*), chains are more stretched out due to the excluded volume effect to support higher normal load and ensure a thin solvent lower at the interface, even at higher compression. This thin solvent layer ensures lower friction at higher compression. The decrease in the friction coefficient with increasing *ρ* was observed in experiments [26], where at a moderate load/pressure, the system with highest grafting density exhibits the lowest friction. As the applied load increases, a “transition load” was observed at each grafting density in the experimental work [26]. Above the transition load, the friction was found to be increasing with the increase in grafting density. Over the range of separations *D* we studied, we did not see any “transition load” in our simulation work.

#### 3.2.3. Effect of Shear Velocity for Two Grafting Densities at Fixed Separation

**Figure 9:**
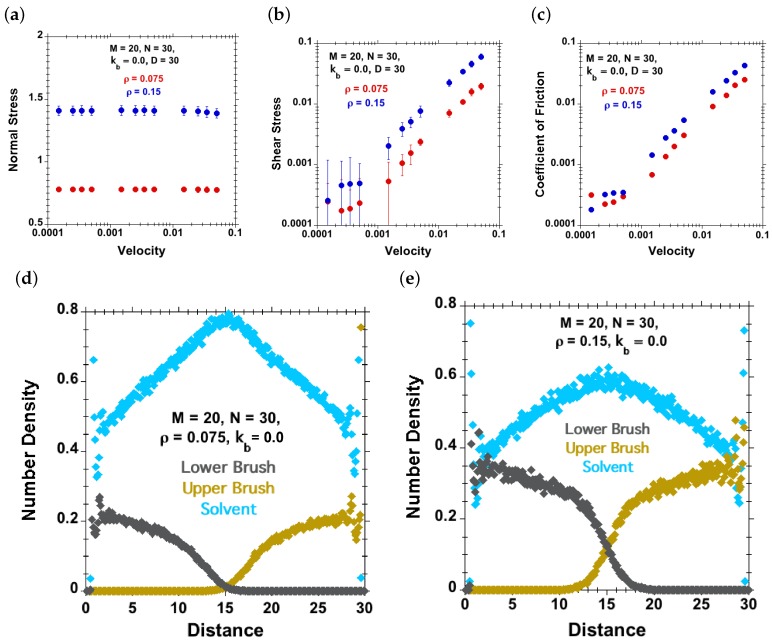
Effect of grafting density (red dots for ρ=0.075 and blue dots for ρ=0.15) for M=20, N=30, $k_{b}=0$, D=30 and good-solvent quality. (**a**) Normal stress against velocity; (**b**) shear stress against velocity; (**c**) coefficient of friction against velocity (Approach B); (**d**–**e**) density profiles of solvent (cyan dots), lower polymer-chains (grey dots) and upper polymer-chains (brown dots) for (**d**) ρ=0.075 and (**e**) ρ=0.15.

Here, normal and shear stresses were calculated for various velocities *v* at a fixed separation D=30. The simulations were performed for systems with different grafting densities ρ=0.075 and ρ=0.15 (Figure 9), but for the same number of grafted chains M=20 on each side having N=30 beads. We observed an increase in normal and shear forces with increasing grafting density at all shear velocities, consistent with an expected greater osmotic pressure, and with a more significant contribution of chain-chain interactions, respectively. In the shear stress versus velocity curve (Figure 9b), there is a slight increase of the shear stress with increasing velocity for brush-brush systems at relatively low velocities. Similar trends were observed in experimental studies [24,25] and are designated as a “boundary-regime”-like behavior. For the corresponding grafting densities and separation (D=30), we have plotted density profiles of solvent and polymer chains. Here, we observe that at lower grafting density (ρ=0.075), the polymer chains are marginally compressed or uncompressed, but at higher grafting density (ρ=0.15) for the same separation (D=30), the brushes are more compressed; and interpenetration between opposite chains is observed, due to larger equilibrium-brush height with increasing grafting density (Figure 2). This higher interpenetration between chains causes higher friction that we observe at higher grafting density. These results are in agreement with Galuschko et al. [20], where an increase in friction with an increase in grafting density at constant separation distance was observed for a brush-against-brush system with a dissipative-particle-dynamics (DPD) thermostat.

To summarize, we explored two different approaches to study the effect of grafting density on the tribological behavior of polymer brushes. In the first approach (A), we kept the separation constant and varied the shear velocity for systems with different grafting densities. In that case, the coefficient of friction was calculated from the ratio between shear and normal stresses at each shear velocity for the different systems. Here, we observed an increase in the coefficient of friction with increasing grafting density at each shear velocity. This increase can be attributed to the fact that more polymers enter the penetration (interaction) zone for a system with higher grafting density. In the second approach (B), we maintained the shear velocity constant, but varied the separation *D* between opposing grafting surfaces for systems with different grafting densities over a range, such that h<D<2h holds. Normal and shear stresses were calculated at each separation. The coefficients of friction were calculated for each system from the slope of the shear stress against normal stress curve. Following this approach, we observed a decrease in friction with increasing grafting density.

**Figure 10:**
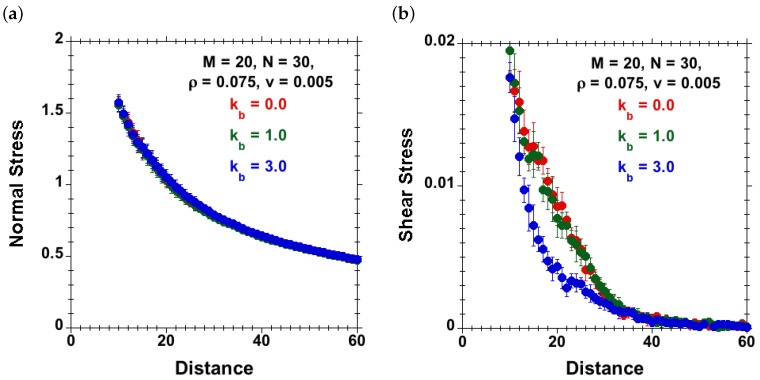
Steady-state (**a**) normal stress and (**b**) shear stress against separation for brush-against-brush systems (M=20, N=30, good-solvent conditions, ρ=0.075) subjected to shear velocity v=0.005 for chains with varying stiffness: $k_{b}=0$ (red), $k_{b}=1$ (green) and $k_{b}=3$ (blue). For intermediate distances, there is a remarkable drop of shear stress for the stiffer chains, while the normal stress remains basically unaffected by $k_{b}$, as in equilibrium (Figure 5).

Here, we would like to make an attempt to rationalize the two different results obtained via A and B for the coefficient of friction. From our simulations, it seems that the normal stress is independent of *v* and increases with decreasing separation distance *D*, i.e., $\sigma_{zz}\left(v,\rho ,D\right)=\sigma_{zz}^{\mathrm{bulk}}+d_{2}\left(\rho \right)/D$, where $\sigma_{zz}^{\mathrm{bulk}}$ would correspond to the bulk value (D→∞), and the force density $d_{2}\left(\rho \right)$ depends on *ρ* alone. The shear stress $\sigma_{xz}$ seems to increase linearly with *v*, $\sigma_{xz}\left(v,\rho ,D\right)=\left[c_{xz}^{\mathrm{bulk}}+d_{1}\left(\rho \right)/D\right]v$, parameterized by coefficients $c_{xz}^{\mathrm{bulk}}$ and $d_{1}\left(\rho \right)$. From Figure 9, we infer that both $d_{1}\left(\rho \right)$ and $d_{2}\left(\rho \right)$ tend to increase with increasing *ρ*, and Figure 10 seems to suggest that $c_{xz}^{\mathrm{bulk}}$ is extremely small or even vanishes. For approach A, the definition of the coefficient of friction is:

(5) $$\begin{array}{ccc} {\mathrm{CoF}}_{A} & = & \frac{\sigma_{xz}}{\sigma_{zz}}=\frac{c_{xz}^{\mathrm{bulk}}D+d_{1}\left(\rho \right)}{\sigma_{zz}^{\mathrm{bulk}}D+d_{2}\left(\rho \right)}\;v \end{array}$$

while for B, we look for a linear relation between $\sigma_{xz}$ and $\sigma_{zz}$ as we vary *D*. Inverting the relation $\sigma_{zz}\left(v,\rho ,D\right)$ for fixed *ρ* and *v* to find $D(\sigma_{zz},v,\rho )$ and inserting into the corresponding expression for $\sigma_{xz}\left(v,\rho ,D\right)$, we obtain such a linear relationship, $\sigma_{xz}=\mathrm{const}.+{\mathrm{CoF}}_{B}\sigma_{zz}$ with:

(6) $$\begin{array}{ccc} {\mathrm{CoF}}_{B} & = & \frac{d_{1}\left(\rho \right)}{d_{2}\left(\rho \right)}\;v \end{array}$$

First of all, ${\mathrm{CoF}}_{A}$ and ${\mathrm{CoF}}_{B}$ cannot be expected to be identical. Our results for approach B have shown that $d_{1}\left(\rho \right)/d_{2}\left(\rho \right)$ tends to decrease with increasing *ρ*, while A revealed an increasing ${\mathrm{CoF}}_{A}$ with increasing *ρ*. Assuming $c_{xz}^{\mathrm{bulk}}=0$, we can rationalize our results if we tentatively approximate $d_{2}\left(\rho \right)\approx \rho d_{1}\left(\rho \right)$ and assume small enough values of $\rho /\sigma_{xz}^{\mathrm{bulk}}D$. For sufficiently strong compression, the bulk contributions become irrelevant, and ${\mathrm{CoF}}_{A}$ and ${\mathrm{CoF}}_{B}$ lead to identical results.

### 3.3. Sheared Brushes Made of Semiflexible Chains

#### 3.3.1. Effect of Chain Stiffness and Separation at Fixed Velocity and Surface Density

The simulations were performed for a brush-against-brush system having specifications, M=20, N=30 and ρ=0.075. Such a model brush system was studied for different chain-stiffness values $k_{b}=0$, 1 and 3. A constant shear velocity v=0.005 was applied on tethered beads on both sides, but moving in opposite directions. The simulations were run over different separations *D*, and stresses were calculated at each *D*. Figure 10a–b shows normal and shear stresses against separation *D* curves obtained for chains with different persistence lengths. We observe a lower shear stress at a given normal stress for systems with chains of higher stiffness. The normal stress versus distance plot does not show any effect of chain stiffness, while the shear stress versus distance graph (Figure 10b) highlights a decrease of the shear stress with increasing chain stiffness at identical separations. This can be explained by the fact that even though at the same separation the system with higher stiffness shows higher interpenetration between opposite chains, brushes having stiffer chains align themselves along the shear direction easily, so the friction decreases rather than increases.

#### 3.3.2. Effect of Velocity and Chain Stiffness at Fixed Separation and Surface Density

**Figure 11:**
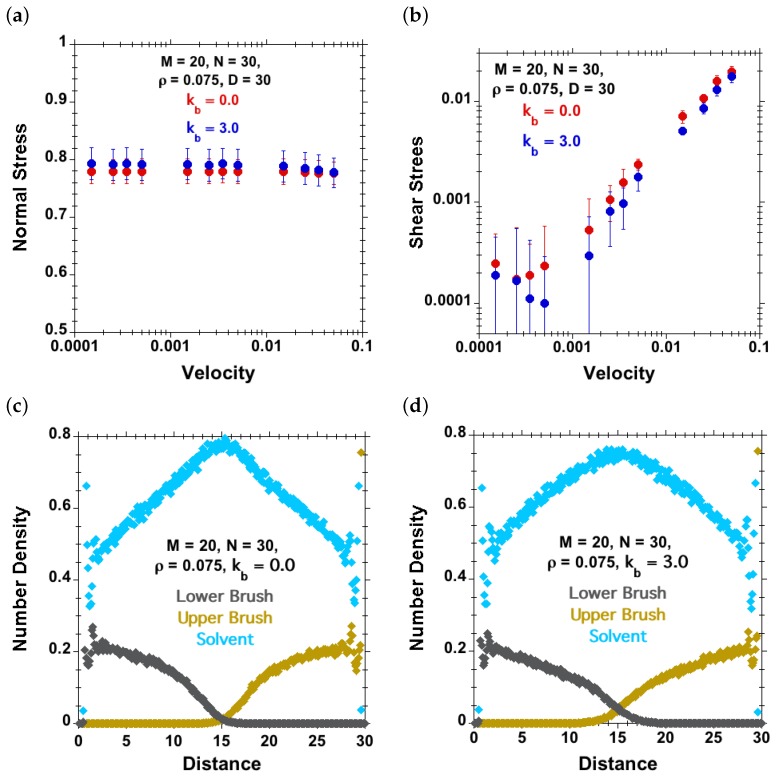
Effect of chain stiffness (red dots for $k_{b}=0$ and blue dots for $k_{b}=3$) for M=20, N=30, ρ=0.075, D=30 and good-solvent quality. (**a**) Normal stress against velocity; (**b**) shear stress against velocity; (**c**–**d**) concentration profiles of solvent (cyan), lower polymer-chains (grey) and upper polymer-chains (brown) for (**c**) $k_{b}=0$ (flexible) and (**d**) $k_{b}=3$ (semiflexible).

Here, normal and shear stresses are plotted against shear velocity at a separation of D=30 and surface density ρ=0.075 for two different values of chain stiffness $k_{b}=0$ and $k_{b}=3$ in Figure 11. We observe an increase in normal stress with the increase in chain stiffness, whereas the shear stress decreases with increasing chain stiffness. These two observations lead to the conclusion that the coefficient of friction decreases with increasing persistence length over the range of stiffness studied. Here, again, we have observed boundary-regime-like behavior [24,25] at lower velocities in the shear stress versus shear velocity representation (Figure 11b). The corresponding density-profile plots show that flexible ($k_{b}=0$) polymer chains are marginally compressed at separation D=30, but for stiffer chains ($k_{b}=3$), interpenetration is observed for the same separation D=30.

### 3.4. Comparing Flexible and Semiflexible Brushes of Identical Height

**Figure 12:**
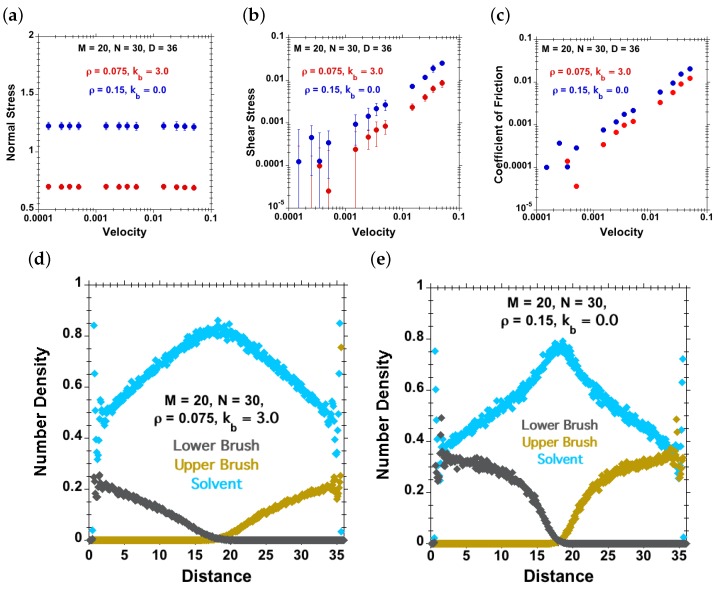
Effect of the combination of chain stiffness and grafting density (red dots for ρ=0.075, $k_{b}=3$ and blue dots for ρ=0.15, $k_{b}=0$) resulting in an equilibrium-brush height h=18 in each case (remaining identical system parameters: M=20, N=30 under good-solvent conditions). (**a**) Normal stress against velocity; (**b**) shear stress against velocity; (**c**) coefficient of friction, as well as the concentration profiles of solvent (cyan dots), lower polymer-chains (grey dots) and upper polymer-chains (brown dots) for (**d**) ρ=0.075 and $k_{b}=3$ and (**e**) ρ=0.15 and $k_{b}=0$.

In Figure 4, we have observed that different combinations of grafting density (*ρ*) and chain stiffness ($k_{b}$) can result in a similar equilibrium-brush height (*h*). This is also clear from Equation (4). For example, two different combinations, first ρ=0.075 and $k_{b}=3$ and second ρ=0.15 and $k_{b}=0$, result in a basically identical equilibrium-brush height, h≈18. In Figure 12a–c, the normal and shear stresses are plotted against shear velocities for these two different cases discussed earlier at the same separation, D=36. The corresponding concentration profiles in Figure 12d–e confirm the similar equilibrium-brush heights. We have observed higher normal and shear stresses at all shear velocities for the combination where the chains are more flexible and the grafting density higher (though the equilibrium-brush height is very comparable in both cases). The height *h* and dimensionless ratio h/D alone do therefore not uniquely determine the tribological properties, but the chain stiffness and grafting density serve as additional design parameters.

## 4. Conclusions

NEMD simulations were performed on a brush-against-brush system with explicit solvent beads. The effect of grafting density and chain stiffness on the tribological behavior of polymer brushes was studied. When the separation between polymer-bearing surfaces is maintained constant and the friction behavior of brushes monitored over different shear velocities, the friction is found to increase with increasing grafting density. However, when the velocity is kept constant and the friction behavior was studied over different separations, the coefficient of friction is found to decrease with increasing grafting density. This can be explained in terms of the load-bearing capacity of polymer brushes with higher grafting density, and we provided some rationale to interpret the differences at weak compression, while we expect agreement between both measures at sufficiently strong compression. The friction coefficient furthermore depends clearly on chain stiffness and is found to decrease with increasing persistence length, both in the velocity- and separation-dependency studies, over the stiffness range explored.

This work was undertaken to help establishing design rules for polymer-brush assisted lubrication and to shed some light on the effect of tuning parameters, such as grafting density and persistence length on the tribological properties of brushes sheared against each other. Semiflexible polymer brushes are particularly relevant in the context of DNA-coated nanoparticle crystallization. A natural progression from this work includes exploring the effect of molecular weight and solvent quality to better understand the interplay of these additional tuning parameters on the tribological behavior of the model polymer brush. The solvent quality can be varied by changing the attractive part of interactions between solvent and polymer beads. Furthermore, constant-load simulations would be helpful to address the experimentally observed transition load.

## Figures and Tables

**Table 1:** Simulation parameters for the separation-dependency studies. To calculate a Weissenberg number Wi $=2\tau_{R}v/D$ at different grafting densities and velocities, we employ the empirical relationship for the relaxation time $\tau_{R}\approx N^{2.31}{\left(D/\rho \right)}^{-0.31}$ [20,59], with N=30.

Grafting density	Velocity range	Separation	Relaxation time	Weissenberg numbers
*ρ*	*v*	*D*	$\tau_{R}$	Wi
0.015	0.001	12–24	325–262	0.054–0.022
0.025	0.001	13–26	371–299	0.056–0.024
0.075	0.001	15–30	499–403	0.066–0.026
0.15	0.001	18–36	585–472	0.066–0.026

**Table 2:** Same as Table 1 for the velocity-dependency studies.

Grafting density	Velocity range	Separation	Relaxation time	Weissenberg numbers
*ρ*	*v*	*D*	$\tau_{R}$	Wi
0.075	0.00015–0.05	20	457	0.00686–2.28
0.075	0.00015–0.05	30	403	0.004–1.34
0.075	0.00015–0.05	40	368	0.0028–0.92
0.15	0.00015–0.05	30	499	0.005–1.66
